# Expression of CIB1 correlates with colorectal liver metastases but not with peritoneal carcinomatosis

**DOI:** 10.1186/s12885-021-08927-w

**Published:** 2021-11-18

**Authors:** Sven Jacob, Florian Bösch, Markus B. Schoenberg, Elise Pretzsch, Christopher Lampert, Ren Haoyu, Bernhard W. Renz, Marlies Michl, Jörg Kumbrink, Thomas Kirchner, Jens Werner, Martin K. Angele, Jens Neumann

**Affiliations:** 1 Department of General, Visceral and Transplantation Surgery, University Hospital, LMU Munich, Munich, Germany; 2 Department of Medicine III, University Hospital, LMU Munich, Munich, Germany; 3 Institute of Pathology, Medical Faculty, Ludwig-Maximilians-University (LMU) Munich, Marchioninistr. 15, 81377 Munich, Germany; 4 German Cancer Consortium (DKTK); German Cancer Research Centre (DKFZ), Heidelberg, Germany

## Abstract

**Background:**

Molecular differences in colorectal cancer (CRC) are associated with the metastatic route. Patient survival is mainly driven by metastatic spread thus it is imperative to understand its key drivers to develop biomarkers for risk stratification, follow-up protocols and personalized therapy. Thus, this study aimed to identify genes associated with the metastatic route in CRC.

**Material and methods:**

CRC patients resected at our clinic from 2005 to 2014 and with a minimum 5-year follow-up were included in this analysis and grouped into CRC with hepatic (HEP), peritoneal (PER) or without distant metastases (M0), and HEP/PER. Firstly, tumor RNA of 6 patients each was isolated by microdissection from formalin-fixed paraffin-embedded specimens and analyzed by a NanoString analysis. Subsequently, these results were validated with immunohistochemistry and correlated to clinicopathological parameters in a larger collective of CRC patients (HEP *n* = 51, PER *n* = 44, M0 *n* = 47, HEP/PER *n* = 28).

**Results:**

Compared to M0, HEP tumors showed 20 differentially expressed genes associated with epithelial-mesenchymal transition (EMT) and angiogenesis. Compared to M0, PER tumors had 18 differentially expressed genes. The finding of different gene signatures was supported by the multidimensional principal component clustering analysis. Tumor perforation did not influence the metastatic route. CIB1 was homogenously and significantly overexpressed in HEP compared to M0 (*p* < 0.001), but not in PER. Furthermore, immunohistochemical validation demonstrated that the mean CIB1 expression in HEP was 80% higher than in M0 (*p* < 0.001).

**Conclusion:**

Gene expression analysis revealed that CIB1 is significantly overexpressed in CRC leading to liver metastases compared to M0 and PER. Thus, the present results suggest that CIB1 may play a crucial role for hematogenous spread to the liver but not for peritoneal carcinomatosis. Consequently, CIB1 seems to be a promising prognostic marker and a potential tool for future targeted therapies as well as early diagnostics and follow-up.

## Introduction

Colorectal cancer (CRC) is the third most common malignancy worldwide and causes annually 600,000 cancer related deaths [1, 2]. Survival is mostly influenced by the development of distant metastases. In this respect, the hematogenous spread to the liver and peritoneal carcinomatosis are the main drivers [3, 4]. Almost a third of patients with CRC develop either synchronous or metachronous metastases to the liver or suffer from peritoneal carcinomatosis [5, 6]. Thus, it is imperative to understand and characterize key drivers of the metastatic route.

In this respect, epithelial-mesenchymal transition (EMT) and angiogenesis play an important role in the metastatic process and organotropism [7–9]. Likewise, the interaction between tumor biology, inflammation and immunology directly influences the formation of distant metastases [10, 11]. Nonetheless, up until now the exact underlying mechanisms of metastasis and organotropism remain ill-defined [12]. Sensitive biomarkers for risk stratification as well as diagnostic tools need to be identified to achieve the goal of precision medicine and develop tailor made personalized therapeutic approaches. Furthermore, individually adapted follow-up protocols might become possible. Therefore, novel diagnostic biomarkers are highly demanded identifying patients at risk for distant metastases.

In an attempt to elucidate the relevance of angiogenesis, EMT and metastasis associated pathways on organotropism, the present study aimed to analyze the expression rates of onco- and tumor suppressor genes associated with different metastatic patterns of CRC. Therefore, gene expression analysis of locally advanced CRC without distant spread, CRC leading to liver metastases, peritoneal carcinomatosis or to both was performed. A multidimensional principal component analysis characterized the individual gene signature of each specific group. Subsequently, the results were validated by immunohistochemistry in a larger cohort of CRC specimen.

## Material and methods

Patients undergoing surgery at the Department of General, Visceral and Transplantation Surgery at the Ludwig-Maximilian University Hospital Munich (Munich, Germany) due to CRC from January 2005 to December 2014 were tabulated. These patients were stratified into four groups: patients with locally advanced CRC without metastases within a five-year follow-up (M0), patients with distant metastases exclusively in the liver, either synchronous or metachronous (HEP), patients with peritoneal carcinomatosis (PER) and patients with both, liver metastases and peritoneal carcinomatosis (HEP/PER). The surgical specimen were classified according to the current WHO 2019 and TNM classification of malignant tumors 2018. Tumor perforation was defined as an undoubtedly perforation of all layers of the intestinal wall leading to a communication of the lumen of the bowel and the peritoneal cavity. All patients were screened for a follow-up period of at least 5 years to guarantee individual group assignment. Clinicopathological information for baseline patient characteristics as well as the corresponding formalin-fixed paraffin-embedded (FFPE) tissue of the primaries were collected for further analysis. Patients presenting a lack of any of these baseline variables or specimens or presenting with co-malignancies, hereditary non-polyposis colon cancer (HNPCC) or familiarly adenomatous polyposis (FAP) were excluded. To generate the four groups a case-control design was applied. Thus, the final panel consisted of 46 patients in M0, 51 in HEP, 44 in PER and 28 in HEP/PER. The study was performed according to the recommendations of the local ethics committee of the Medical Faculty of the LMU Munich who approved the protocol of the study (no. 19–966). Irreversibly anonymized data sets and specimens were used for this study. Research was performed according to the standards laid out in the declaration of Helsinki 1975. All researchers were blinded to the patient data during the experimental analysis.

### Gene expression analysis in the baseline cohort

In a first step, six patients of each group were randomly selected for gene expression analysis, respectively (Table 1). An experienced pathologist (JN) marked tumor-containing areas on hematoxylin and eosin-stained histological serial sections. The marked areas were microdissected under microscopic control using scalpel-blades. From the resulting tissue, RNA was isolated using the RNeasy FFPE® kit (Qiagen, Hilden, Germany) following the users handbook. RNA yield and quality (260/280 absorbance ratio) were assessed using the NanoDrop ND-1000 spectrophotometer (NanoDrop Technologies, Rockland, USA). mRNA expression was measured with the NanoString nCounter FLEX Analysis System (NanoString Technologies, Seattle, USA) using 100 ng of total RNA. The PanCancer Progression Panel CodeSet for 770 genes including 30 reference genes was hybridized to total RNA for 18 h at 65 °C. The expression data were analyzed utilizing the NanoString nSolver Analysis Software v4.0. Quality control and normalization of the data was performed using the default settings and algorithm within the nSolver software and by analyzing the positive and negative controls, housekeeper reference genes and total counts (excluding controls) as well as the binding densities in each sample.

**Table 1:** Baseline patient characteristic: gene expression cohort

Variable	M0 n (%)	HEP n (%)	PER n (%)	***p***-value
**Patients**	6	6	6	
**Medium age (y)**	71	69	71	0.72
**Sex**				
Female	5 (83.0)	4 (47.0)	4 (52.5)	0.75
Male	1 (17.0)	2 (33.3)	2 (33.3)	
**Location of PT**				
Right colon	2 (33.3)	2 (33.3)	4 (66.7)	0.40
Left colon	4 (66.7)	4 (66.7)	2 (33.3)	
**UICC**				> 0.9
I	0 (0.0)	0 (0.0)	0 (0.0)	
II	0 (0.0)	0 (0.0)	0 (0.0)	
III	6 (100.0)	1 (0.17)	0 (0.0)	
IV	0 (0.0)	5 (99.82)	6 (100.0)	
**Grading**				
Low grade	3 (50.0)	3 (50.0)	3 (50.0)	0.99
High grade	3 (56.9)	3 (50.0)	3 (50.0)	
**Time of liver metastases**				
Synchron	n.a.	5 (83.0)	n.a.	n.a.
Metachron	n.a.	1 (17.0)	n.a.	
**Chemotherpy**				
Yes	6 (100.0)	6 (100.0)	6 (100.0)	> 0.9
No	0 (0.0)	0 (0.0)	0 (0.0)	

*UICC* union for international cancer control

### Validation of baseline results via immunohistochemistry

In a second step, the results of the baseline cohort analysis were validated via immunohistochemistry in a larger cohort of patients with CRC distinguished by their metastatic behavior (Table 2). For Calcium and Integrin-binding Protein 1 (CIB1) immunohistochemistry, 5 μm standard tissue sections of the FFPE specimens were stained using a CIB1 rabbit polyclonal antibody (Atlas Antibodies, Stockholm, Sweden). Slides were subjected to heat-induced epitope retrieval using Pro Taqs V Antigen Enhancer (Quartett, Berlin, Germany). Primary antibodies (dilution of 1:80) were incubated for 1 h at RT. The slides were then washed and subsequently developed by adding the detection-system ImmPRESS Anti-Rabbit IgG Polymer Kit (Vector Laboratories, Burlingame, CA). The DAB+ system (Agilent Technologies, Santa Clara, CA) was used as chromogen and the slides were then counterstained with Hematoxylin Gill’s Formula (Vector Laboratories, Burlingame, CA). To exclude non-specific staining, isotype and system controls were included.

**Table 2:** Baseline patient characteristic: validation cohort

Variable	M0 n (%)	HEP n (%)	PER n (%)	HEP/PER n (%)	***p***-value
**Patients**	47	51	44	28	
**Medium age (y)**	66	65	67	61	0.55
**Sex**					
Female	20 (44.0)	24 (47.0)	23 (52.5)	13 (46.0)	0.36
Male	27 (56.0)	27 (53.0)	21 (47.5)	15 (54.0)	
**Location of PT**					
Right colon	15 (27.8)	15 (28.9)	17 (38.5)	9 (31.6)	0.80
Left colon	32 (72.2)	36 (71.1)	27 (61.5)	17 (68.4)	
**UICC**					
I	17 (36.0)	1 (2.9)	0 (0.0)	0 (0.0)	0.00011
II	11 (23.5)	3 (5.9)	4 (9.0)	2 (7.1)	
III	19 (40.5)	6 (1.8)	14 (31.0)	4 (14.3)	
IV	0	41 (89.4)	26 (60.0)	22 (78.6)	
**Grading**					
Low grade	29 (61.7)	29 (56.9)	22 (50.0)	17 (60.8)	0.70
High grade	18 (38.3)	22 (44.1)	22 (50.0)	11 (39.2)	
**Time of liver metastases**					
Synchron	n.a.	41 (89.4)	n.a.	22 (78.6)	0.5
Metachron	n.a.	10 (10.6)	n.a.	6 (22.4)	
**Tumor perforation**					
Yes	0 (0.0)	3 (5.9)	4 (9.1)	10 (35.7)	0.0002
No	47 (100)	48 (94.1)	40 (90.9)	18 (64.3)	

*UICC* union for international cancer control, *T* tumor size, *N* lymph node status, *n.a.* not applicable

### Scoring of immunohistochemistry

**Fig. 1:**
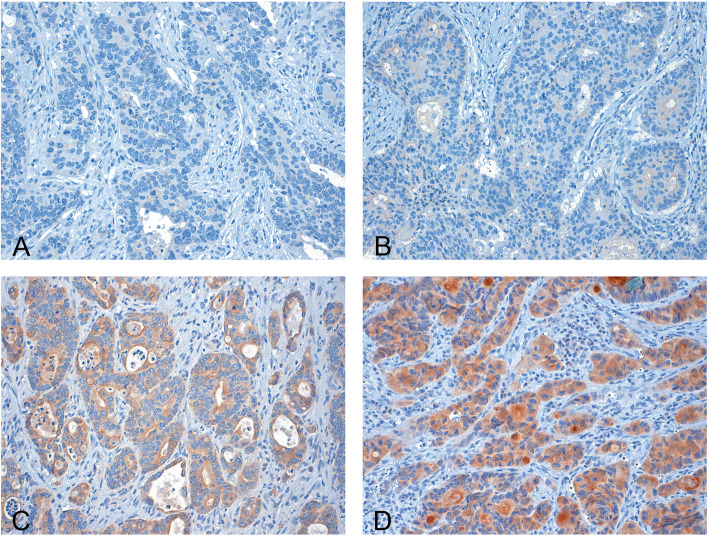
Immunohistochemical staining of CIB-1: Colonic adenocarcinoma with absent (**A**), weak (**B**), moderate (**C**) and strong (**D**) CIB-1 staining (magnification 200-fold)

Two independent and blinded observers (S.J. and J.N.) analyzed the stained tissue samples. Discrepant cases were jointly reviewed under a multiheaded microscope and a consensus was reached. For each region of the tumor an intensity score from 0 to 3 was assigned (0 indicates no staining (Fig. 1A), 1 a weak expression (Fig. 1B), 2 a moderate expression (Fig. 1C) and 3 a strong expression (Fig. 1D). Furthermore, the proportion of the tumor staining for each intensity was recorded as 5% increments from a range of 0–100. A final score (H-score, ranging from 0 to 300) was obtained by adding the sum of scores obtained for each intensity and proportion of area stained. Cases showing an H-score above the median (median = 105) were categorized as high-grade expression, whereas cases with a lower score were classified as low-grade expression [13].

### Statistical analysis

The baseline gene expression values acquired by the nCounter® PanCancer Progression Panel by NanoString-Technologies (Hamburg, Germany) were tabulated. Gene signatures of M0, HEP and PER were explored by applying a multidimensional principal component analysis (PCA). Missing input data was calculated using the missing forest algorithm. Next, gene expression rates were characterized comparing M0 vs. HEP and M0 vs. PER by one-way ANOVA. Differential expression was presented in volcano plots illustrating the level of significance and fold change. Significantly differentially expressed genes were plotted in heat maps. *P*-values < 0.05 were considered significant, *p*-values < 0.001 highly significant.

Correlations of the immunohistochemical validation analyses as well as clinicopathological features were tested using a two-sided χ2-test, students T-tests and one-way Anova. Statistical analyses and illustrations were performed using SPSS v 20.1 for Mac (IBM Corp., Armonk, NY) and Prism 8.0 for Mac (GraphPad Software, Inc., La Jolla, CA) as well as RStudio (Version 3.2.6, RStudio Inc., USA) and the NanoString Technology software package nSolver4.0 (2017).

## Results

### Clinicopathological parameters

Clinicopathological parameters according to the current WHO 2019 and TNM classification of malignant tumors 2018 [14, 15] of the baseline cohort for gene expression analysis and the validation cohort are presented in Tables 1 and 2, respectively. In total, 170 patients were included in the present study. Of these, 47 were classified as M0, 51 as HEP, 44 as PER and another 28 as HEP/PER. To attain homogenous groups, the minimum follow-up period was 5 years. There was no statistical difference in the medium age within the analyzed groups. Of all patients, 80 were female and 90 were male, equally distributed in the four subgroups. In each subgroup, the majority pf primary tumors were found in the left colon with no significant difference among the four groups (*p* >  0.05). In the subgroup including patients who exhibit liver metastases as well as peritoneal carcinomatosis (HEP/PER), 36% for tumors displayed a perforation of the serosa. In PER only 9% had a perforated tumor, whereas in HEP and M0 the perforation rate was 6 and 0%, respectively.

### Gene expression analysis of the baseline cohort

**Fig. 2:**
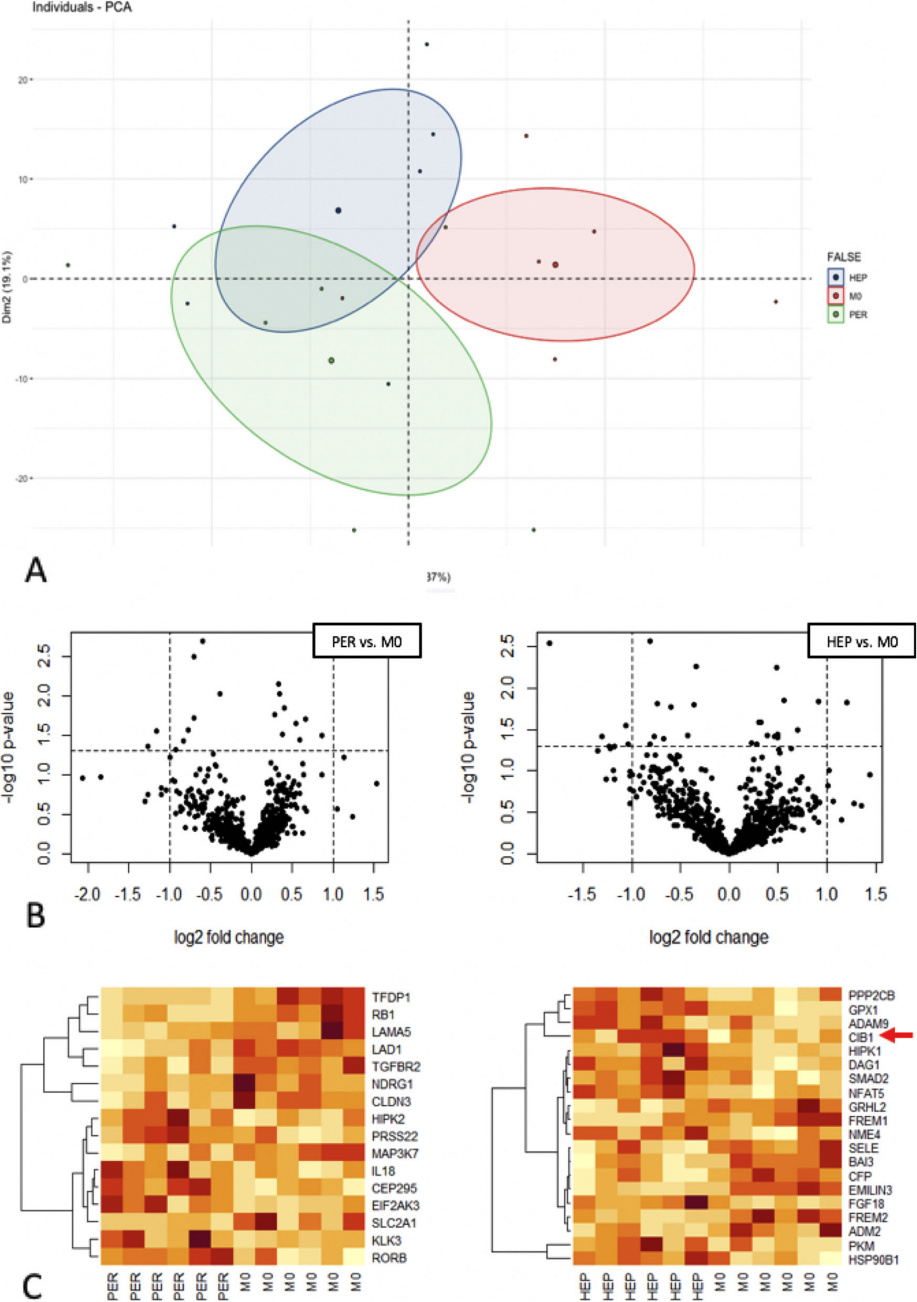
Gene expression presented in a principal component analysis (**A**); volcano plots (**B**); heat maps (**C**): dark red = strong expression, orange = semi-strong expression, white = weak expression

In order to investigate the linear gene expression values of M0, HEP and PER obtained by the NanoString analysis a multidimensional, cluster-analyzing PCA was used. The PCA biplot in Fig. 2A displays distinctly different gene signatures when comparing the clustering of gene expression rates of HEP, M0 and PER with only a marginal point of intersection. The volcano plots in Fig. 2B illustrate each gene of HEP and PER according to its level of significance and log2 fold change when compared to M0. This analysis further supports the PCA. Twenty genes were significantly differentially expressed in HEP compared to M0 (*p* < 0.05). The log2 fold change ranged from − 3.58 to 2.31. In PER, 18 genes demonstrated significant different expression rates with a log2 fold change ranging from − 2.4 to 1.82 (*p <* 0.05). All significantly differentially expressed genes are presented in the heat map in Fig. 2C. With a minimum of 1769.57 and a maximum of 2557.66, CIB1 was homogenously overexpressed in HEP compared to M0 (*p* < 0.02, standard deviation 322.2). CIB1 overexpression is well known in CRC to be associated with deteriorated survival rates [16]. Moreover, it was the most homogeneously overexpressed gene in HEP compared to M0 and PER, thus the CIB1 expression rates were further validated by immunohistochemistry.

### Immunohistochemical validation of CIB-1 expression

**Fig. 3:**
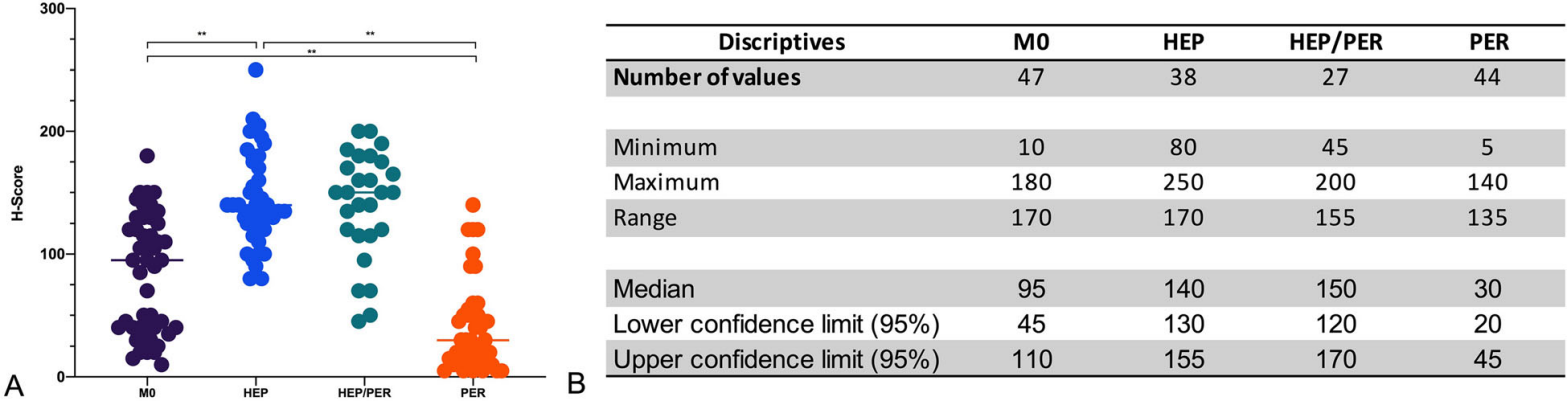
Distribution pattern of immunohistochemical CIB1 expression in CRC patients with different metastatic routes. **A**: One-way ANOVA of all four groups. **B**: Summary of descriptive statistics. **p-value< 0.001

**Fig. 4:**
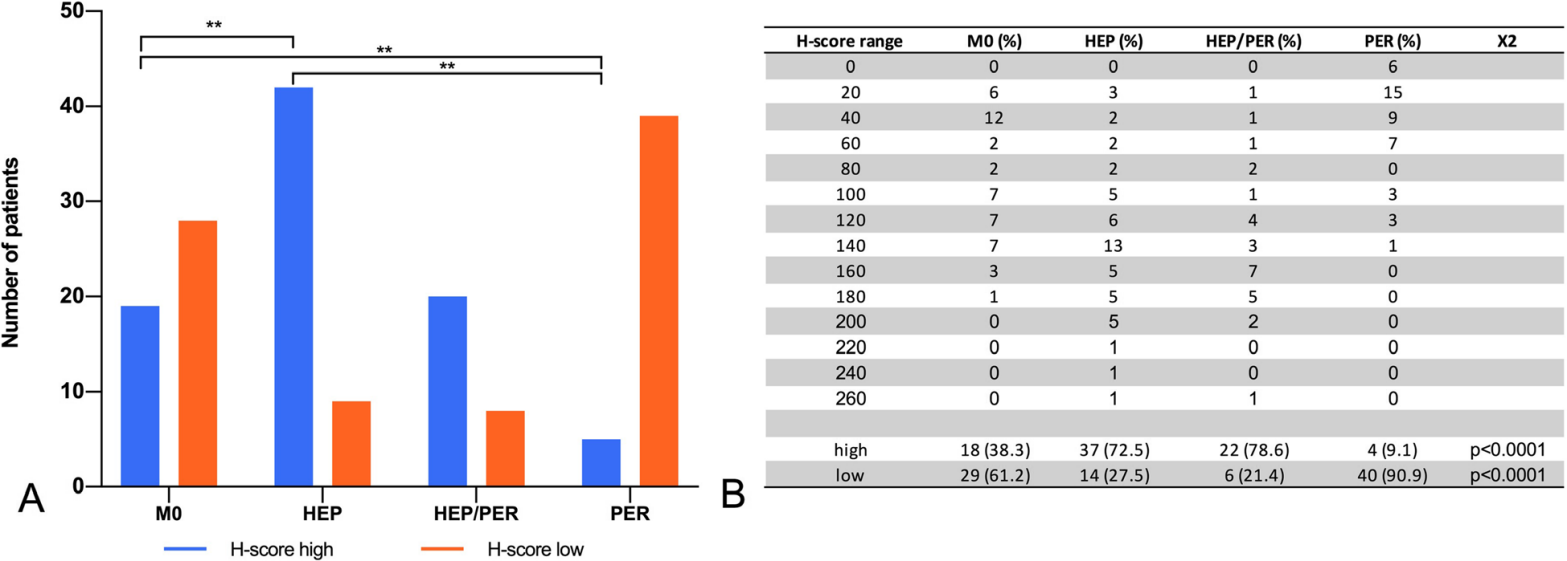
Categorization of CIB1 expression: high vs. low. **A**: Interleaved bars comparing CIB1 category in each subgroup. **B**: H-score range and high vs. low rating. **p-value< 0.001

Immunohistochemical validation revealed a significant correlation of increased CIB-1 expression levels with liver metastases, but there was no significant correlation with peritoneal carcinomatosis. In M0, the mean CIB1 H-score was 83.1. In contrast, HEP patients had a significantly higher mean CIB1 H-score of 145 (*p* < 0.001). The CIB1 H-score (40.23) of PER patients was significantly lower compared to M0 and HEP (*p* < 0.005), respectively (Fig. 3). Consequently, the CIB1 immunoscore in HEP was classified as “high” in 72.5%, compared to 38.3 and 9.1% in M0 and PER, respectively (*p* < 0.0001) (Fig. 4). Furthermore, the sequence of metastasis formation did not correlate with the CIB1 immunoscore. The immunoscore of synchronously metastasized patients was “high” in 54.8% compared to 42.9% in metachronously metastasized patients (*p* >  0.05).

Since patients with mucinous adenocarcinoma have a significantly worse prognosis and decreased disease-free survival rates the primary tumors were classified according to their growth pattern in mucinous (*n* = 38; 22.4%) and non-mucinous tumors (*n* = 132; 77.6%) [17, 18]. Eleven mucinous tumors (29%) had high CIB1 expression levels compared to 67 non-mucinous tumors (50.8%) (*p* < 0.05).

## Discussion

Colorectal cancer without distant metastases is a frequently overserved and fairly treatable disease in western countries [1, 2]. However, the clinical course of locally advanced CRC is not predictable and although patients might have similar tumor stages, the clinical courses differ widely. Patients might develop metastases either to the liver, the brain, the lungs, or the peritoneum, but there are also patients who will not metastasize. These different courses are highly intriguing from a clinical and mechanistic viewpoint since it may allow to define potential mediators associated with the process of metastatic spread. Thus, the present study aimed to determine the expression rates of tumor suppressor genes and oncogenes in the primary tumor of CRC and to correlate the identified genes with the metastatic route. Therefore, the present study analyzed different collectives distinguished by their metastatic route. After a 5-year follow up, the primary tumors were analyzed in a first step with the “PanCancer Progression Panel” from NanoString. This panel has been shown previously to comprehensively characterize genes involved in tumor progression and metastasis of solid tumors [19, 20]. Subsequently, the multidimensional PCA revealed a distinct expression pattern of the analyzed genes throughout the defined groups and the most homogenously expressed gene was CIB1.

In this respect, CIB1 was validated via immunohistochemistry in the entire cohort to determine its impact on the metastatic route of CRC. CIB1 is a small intracellular and ubiquitously expressed EF-hand calcium binding protein [21]. With the ability of binding soluble as well as transmembrane proteins, it is involved in a variety of cellular processes such as calcium signaling, survival, migration, adhesion and apoptosis [16]. CIB1 is also involved in the development and progression of various malignancies [22–24]. Regarding CRC, CIB1 induces tumor progression by promoting higher levels of vascular endothelial growth factor (VEGF) via Protein kinase D2 (PKD2) and thus is directly involved in angiogenesis [23, 25, 26]. Furthermore, CIB1 mediates its oncogenic potential via antiapoptotic measures through sphingosein kinase 1 (SK1) and nuclear factor kappa-light-chain-enhancer of activated B-cells (NF-κB) [23, 25, 26]. Also, it takes part in sustaining cancer cell viability via Pi3K/AKT and MEK/ERK pathways [23, 25, 26].

The NanoString analysis of tumors leading to liver metastases demonstrated a strong overrepresentation of CIB1 on an RNA-basis compared to patients without distant metastases. Furthermore, immunohistochemical validation of CIB1 expression profiles in FFPE specimens from M0, HEP and PER tumors further supported the significantly different expression rates of CIB1 on the protein level in the entire study collective. Previously, it has been shown that oncogenic KRAS mutations bear a profound metastatic potential in CRC patients and correlate with an upregulation of CIB1 expression rates. Moreover, these studies suggest that the oncogenic potential of Ras might be mediated via CIB1 [27, 28].

In addition, CIB1 expression was significantly lower in PER compared to HEP. This is further supported by the fact that the majority of mucinous tumors, which are associated with peritoneal carcinomatosis but not with liver metastasis [18] have significantly lower CIB1 expression levels. The vast majority of tumors (72.2%) exhibiting peritoneal carcinomatosis were classified as T4. It could be argued that peritoneal carcinomatosis is due to locally aggressive tumor growth and subsequent perforation of the serosa. In this regard, there is evidence that the metastatic process is influenced partially by inflammatory cells, cytokines and mediators [29]. Thus, tumor perforation might lead to abdominal infection and increases the peritoneal implantation potential. However, the present findings suggest that genetic factors in the tumor determine organotropism. Accordingly, tumor perforation was found in the minority of patients with either hepatic metastases (9%) or peritoneal carcinomatosis (6%). The concept of organotropism is further supported by the fact that 36% of tumors leading to liver metastases and peritoneal carcinomatosis (HEP/PER) exhibit a perforation of the serosa in the post-operative pathological examination. Thus, it can be assumed that the dissemination to the liver is at least in part mediated by an overexpression of CIB1. Nonetheless, there is no association of the CIB1 expression levels and the rate of primary tumor perforation. Consequently, CIB1 might not only be a significant marker for the presence of liver metastases but also potentially be a convenient biomarker for screening for metachronous liver metastases. In this respect, CIB1 can reliably be determined in urine samples of patients by enzyme linked immunosorbent assays (ELISA) [30]. Therefore, future studies are highly demanded elucidating the clinical impact of CIB1 in the follow-up of CRC patients.

Downstream factors from CIB1 were beyond the scope of the present study. Nonetheless, it seems that CIB1 targets SK1, a central downstream target of Ras. CIB1 is responsible for the translocation of SK1 to the extracellular membrane and thus develop the SK1-product sphingosine1-phosphate (S1P) [26]. Recent evidence suggests that S1P influences the metastatic behavior of many abdominal tumors. In this respect, patients with lung metastases had significantly increased S1P serum levels. Additionally, the administration of the anti-S1P antibody Sphingomab (LPath Inc., San Diego, CA, USA) resulted in a significant reduction of their metastatic pulmonary burden and reduced circulating serum S1P-levels [31]. This might present a possibility to derive a liquid biopsy for screening for CRC metastases in further studies. However, a compound directly targeting CIB1 is currently not available.

To the best of our knowledge, the present study highlights the role of CIB1 in colorectal cancer for the first time. Moreover, this is the first study analyzing the differences of CIB1 expression levels depending on the metastatic route. Although the present results suggest an important role for CIB1 in the metastatic route of CRC patients, there are limitations due to the retrospective character. The study concept was to determine the metastatic route, not only in synchronous but also in metachronous metastasized tumors and to compare those to CRC without distant spread. This concept required an observation period of at least 5 years, which can only be addressed in a retrospective approach. Moreover, the present analysis investigated only 169 patients with CRC, which is one of the most common cancers worldwide. Thus, the results of the present study have to be interpreted carefully. Additionally, a majority of patients had synchronous disease, which might add a bias to the present analysis. In this respect, referred patients frequently have advanced disease since our university hospital has been certified with the highest possible degrees for colorectal as well as for liver surgery. Thus, patients with stage I or II colorectal cancer are commonly not referred to our center. Nonetheless, there was no significant different CIB1 expression levels between patients with synchronous or metachronous metastases.

## Conclusions

The present study demonstrated a significant overexpression of CIB1 in CRC leading to liver metastases compared to patients without distant metastases or peritoneal carcinomatosis. Even in the presence of tumor perforation, CIB1 was associated with hepatic metastases. These results indicate that CIB1 may play a crucial role for metastasis formation to the liver but not for the development of peritoneal carcinomatosis. Moreover, CIB1 might identify patients at risk for metachronous liver metastases in future studies.

## Data Availability

The datasets used and/or analyzed during the current study available from the corresponding author on reasonable request.

## Abbreviations

CIB1: Calcium and Integrin-binding Protein 1
CRC: Colorectal cancer
ELISA: Enzyme linked immunosorbent essays
FAP: Familiarly adenomatous polyposis
FFPE: Formalin-fixed paraffin-ebedded tissue
HE: Hematoxylin and eosi
HNPCC: Hereditary non-polyposis colon cancer
NF-κB: Nuclear factor kappa-light-chain-enhancer of activated B-cells
PCA: Principal Component Analysis
PKD2: Protein Kinase D2
RNA: Ribonucleic acid
SK1: Sphingosine kinase 1
UICC: Union Internationale contre le cancer
VEGF: Vascular-endothelial growth-factor
WHO: World Health Organization
